# 2-Hydr­oxy-5-nitro­benzamide

**DOI:** 10.1107/S1600536809050119

**Published:** 2009-11-28

**Authors:** Abdul Rauf Raza, M. Nawaz Tahir, Bushra Nisar, Mohammad Danish, Mohammad S. Iqbal

**Affiliations:** a Department of Chemistry, University of Sargodha, Sargodha, Pakistan; b Department of Physics, University of Sargodha, Sargodha, Pakistan; c Department of Chemistry, Government College University, Lahore, Pakistan

## Abstract

In the title compound, C_7_H_6_N_2_O_4_, an intra­molecular O—H⋯O hydrogen bond generates an *S*(6) ring. In the crystal, inversion dimers linked by pairs of N—H⋯O hydrogen bonds occur. Weak C—H⋯O links consolidate the packing, leading to *R*_2_^1^(7) and *R*_2_^2^(10) loops within (100) polymeric sheets.

## Related literature

For related structures, see: Pertlik (1990 ▶); Raza *et al.* (2009 ▶).
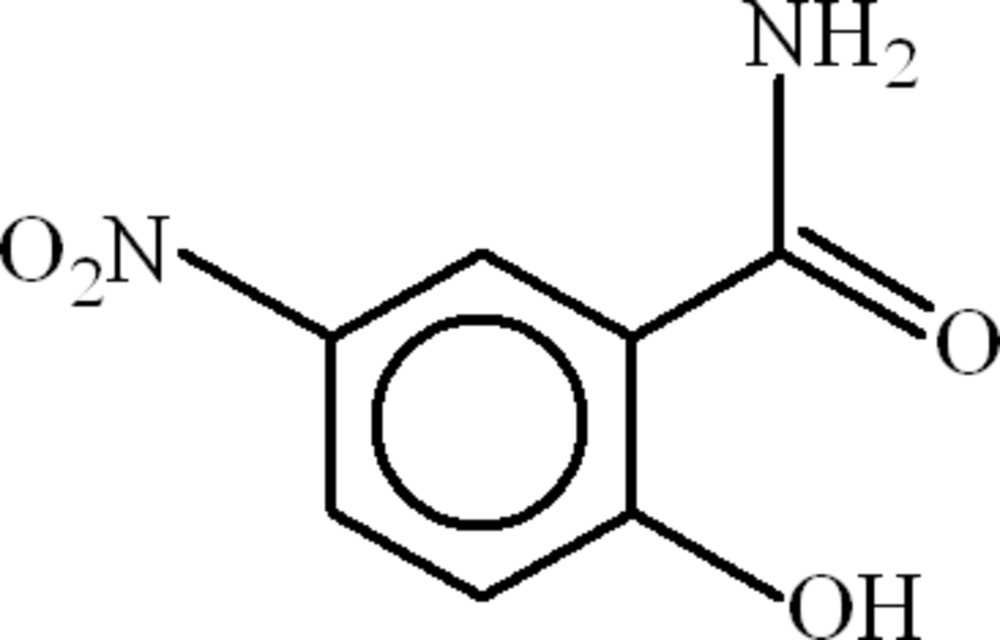


## Experimental

### 

#### Crystal data

C_7_H_6_N_2_O_4_
*M* *_r_* = 182.14
Monoclinic, 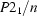

*a* = 5.1803 (3) Å
*b* = 11.1037 (8) Å
*c* = 13.7214 (10) Å
β = 100.642 (4)°
*V* = 775.69 (9) Å^3^
*Z* = 4
Mo *K*α radiation
μ = 0.13 mm^−1^
*T* = 296 K
0.28 × 0.20 × 0.18 mm

#### Data collection

Bruker Kappa APEXII CCD diffractometer
Absorption correction: multi-scan (*SADABS*; Bruker, 2005 ▶) *T*_min_ = 0.970, *T*_max_ = 0.976
4581 measured reflections
1799 independent reflections
1434 reflections with *I* > 2σ(*I*)
*R*_int_ = 0.018

#### Refinement

*R*[*F* ^2^ > 2σ(*F* ^2^)] = 0.037
*wR*(*F* ^2^) = 0.112
*S* = 1.05
1799 reflections
125 parameters
H atoms treated by a mixture of independent and constrained refinement
Δρ_max_ = 0.23 e Å^−3^
Δρ_min_ = −0.21 e Å^−3^

### 

Data collection: *APEX2* (Bruker, 2007 ▶); cell refinement: *SAINT* (Bruker, 2007 ▶); data reduction: *SAINT*; program(s) used to solve structure: *SHELXS97* (Sheldrick, 2008 ▶); program(s) used to refine structure: *SHELXL97* (Sheldrick, 2008 ▶); molecular graphics: *ORTEP-3* (Farrugia, 1997 ▶) and *PLATON* (Spek, 2009 ▶); software used to prepare material for publication: *WinGX* (Farrugia, 1999 ▶) and *PLATON*.

## Supplementary Material

Crystal structure: contains datablocks global, I. DOI: 10.1107/S1600536809050119/hb5244sup1.cif

Structure factors: contains datablocks I. DOI: 10.1107/S1600536809050119/hb5244Isup2.hkl

Additional supplementary materials:  crystallographic information; 3D view; checkCIF report

## Figures and Tables

**Table 1:** Hydrogen-bond geometry (Å, °)

*D*—H⋯*A*	*D*—H	H⋯*A*	*D*⋯*A*	*D*—H⋯*A*
O1—H1⋯O2	0.82	1.79	2.5196 (16)	148
N1—H1*A*⋯O2^i^	0.914 (19)	1.969 (19)	2.8807 (17)	174.9 (18)
N1—H1*B*⋯O3^ii^	0.88 (2)	2.167 (19)	3.0193 (17)	164.6 (15)
C4—H4⋯O1^iii^	0.93	2.49	3.3915 (18)	164
C6—H6⋯O3^ii^	0.93	2.47	3.3826 (16)	169

Symmetry codes: (i) 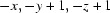; (ii) 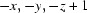; (iii) 
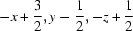
.

**Fig. 1.**
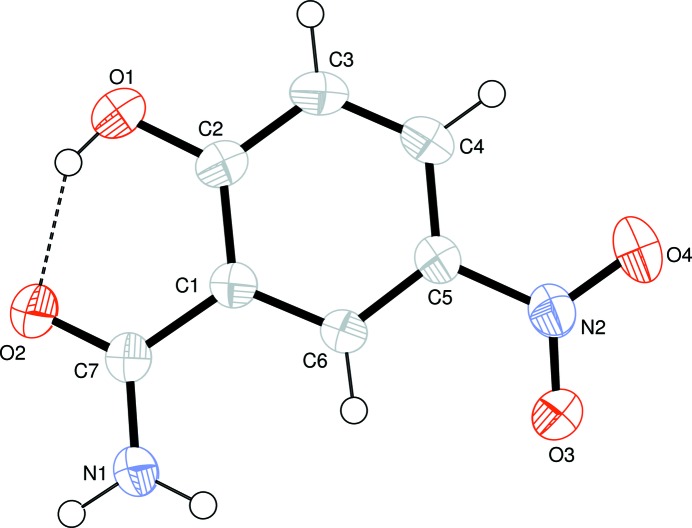
View of (I) with displacement ellipsoids drawn at the 50% probability level. The dashed line represents the intramolecular H-bond.

**Fig. 2.**
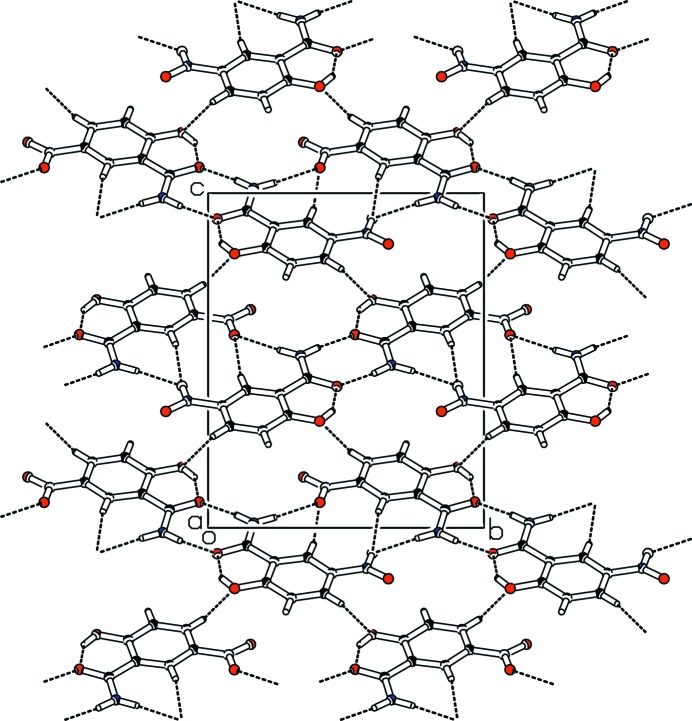
The projectional view of the title compound showing that molecules are dimerized and dimers are linked in the formation of two dimensional polymeric sheets with various ring motifs.

## supplementary crystallographic information

### Comment

The title compound (I, Fig. 1) is an intermediate for various derivatives. We have reported the preparation and crystal structure of (II) 2-hydroxy-3-nitrobenzamide (Raza *et al.*, 2009) which is isomer of (I). The crystal structures of (III) 2-Hydroxybenzamide (Pertlik, 1990) has been published also.

In the asymmetric unit of (I), the benzene ring A (C1—C6) is of course planar. The nitro group B (N2/O3/O4) and the amide group C (C7/N1/O2) make dihedral angle of 8.49 (13)° and 8.48 (21)° respectively, with the benzene ring. The dihedral angle between B/C is 14.51 (22)°. There exist an intramolecular H-bonding of O–H···O type forming S(6) ring motif (Bernstein *et al.*, 1995). The molecules of the title compound are dimerised forming a R_2_^2^(10) and two R_2_^1^(7) ring motifs (Table 1, Fig. 2). The dimers are interlinked each other forming polymeric network and the dimers are surounded by six R_5_^4^(16) ring motifs.

### Experimental

A solution of 2-hydroxy-benzamide (1.37 g, 0.01 mol) in ethyl acetate (25 ml) was added as drops to a nitrating mixture of HNO_3_ (3 ml, 1.89 g, 0.03 mol) and H_2_SO_4_ (2 ml, 1.96 g, 0.02 mol), with constant stirring, while the temperature was kept below 278 K. The reaction mixture was stirred at room temperature for 4–5 h, refluxed for 1 h, cooled, neutralized with aqueous NaHCO_3_ (10%) and extracted with EtOAc (3 × 25 ml). The organic layer was combined, dried over anhydrous Na_2_SO_4_, filtered and rotary concentrated to afford light yellowish solid. The column chromatographic purification with 0, 2.5, and 5% EtOAc in petrol (0.5 l each) over a silica gel packed column (25.5 cm height) afforded the title compound.

### Refinement

The coordintes of H-atoms of NH_2_ group were refined. The other H-atoms were positioned geometrically (O–H = 0.82, C–H = 0.93 Å) and refined as riding with *U*_iso_(H) = 1.2*U*_eq_(C, N, O).

### Crystal data

**Table:**

C_7_H_6_N_2_O_4_	*F*(000) = 376
*M**_r_* = 182.14	*D*_x_ = 1.560 Mg m^−^^3^
Monoclinic, *P*2_1_/*n*	Mo *K*α radiation, λ = 0.71073 Å
Hall symbol: -P 2yn	Cell parameters from 1799 reflections
*a* = 5.1803 (3) Å	θ = 2.4–27.8°
*b* = 11.1037 (8) Å	µ = 0.13 mm^−^^1^
*c* = 13.7214 (10) Å	*T* = 296 K
β = 100.642 (4)°	Prisms, light yellow
*V* = 775.69 (9) Å^3^	0.28 × 0.20 × 0.18 mm
*Z* = 4	

### Data collection

**Table:**

Bruker Kappa APEXII CCD diffractometer	1799 independent reflections
Radiation source: fine-focus sealed tube	1434 reflections with *I* > 2σ(*I*)
graphite	*R*_int_ = 0.018
Detector resolution: 7.50 pixels mm^-1^	θ_max_ = 27.8°, θ_min_ = 2.4°
ω scans	*h* = −6→6
Absorption correction: multi-scan (*SADABS*; Bruker, 2005)	*k* = −14→13
*T*_min_ = 0.970, *T*_max_ = 0.976	*l* = −17→16
4581 measured reflections	

### Refinement

**Table:**

Refinement on *F*^2^	Primary atom site location: structure-invariant direct methods
Least-squares matrix: full	Secondary atom site location: difference Fourier map
*R*[*F*^2^ > 2σ(*F*^2^)] = 0.037	Hydrogen site location: inferred from neighbouring sites
*wR*(*F*^2^) = 0.112	H atoms treated by a mixture of independent and constrained refinement
*S* = 1.05	*w* = 1/[σ^2^(*F*_o_^2^) + (0.0629*P*)^2^ + 0.0918*P*] where *P* = (*F*_o_^2^ + 2*F*_c_^2^)/3
1799 reflections	(Δ/σ)_max_ < 0.001
125 parameters	Δρ_max_ = 0.23 e Å^−^^3^
0 restraints	Δρ_min_ = −0.21 e Å^−^^3^

### Special details

**Table:**

Geometry. Bond distances, angles *etc*. have been calculated using the rounded fractional coordinates. All su's are estimated from the variances of the (full) variance-covariance matrix. The cell e.s.d.'s are taken into account in the estimation of distances, angles and torsion angles
Refinement. Refinement of *F*^2^ against ALL reflections. The weighted *R*-factor *wR* and goodness of fit *S* are based on *F*^2^, conventional *R*-factors *R* are based on *F*, with *F* set to zero for negative *F*^2^. The threshold expression of *F*^2^ > σ(*F*^2^) is used only for calculating *R*-factors(gt) *etc*. and is not relevant to the choice of reflections for refinement. *R*-factors based on *F*^2^ are statistically about twice as large as those based on *F*, and *R*- factors based on ALL data will be even larger.

### Fractional atomic coordinates and isotropic or equivalent isotropic displacement parameters (Å^2^)

**Table:**

	*x*	*y*	*z*	*U*_iso_*/*U*_eq_	
O1	0.5646 (2)	0.40418 (9)	0.31899 (9)	0.0527 (4)	
O2	0.2337 (2)	0.46688 (9)	0.42269 (9)	0.0538 (4)	
O3	0.1375 (2)	−0.09501 (9)	0.42368 (9)	0.0604 (4)	
O4	0.4447 (2)	−0.15349 (9)	0.34804 (9)	0.0599 (4)	
N1	0.0539 (3)	0.33482 (11)	0.51407 (10)	0.0505 (4)	
N2	0.3188 (2)	−0.07405 (10)	0.37931 (8)	0.0425 (4)	
C1	0.3227 (2)	0.26057 (11)	0.39880 (9)	0.0352 (3)	
C2	0.5024 (3)	0.28970 (12)	0.33611 (10)	0.0393 (4)	
C3	0.6220 (3)	0.19813 (14)	0.28969 (11)	0.0460 (4)	
C4	0.5642 (3)	0.07915 (13)	0.30371 (10)	0.0427 (4)	
C5	0.3840 (2)	0.05179 (11)	0.36410 (9)	0.0363 (4)	
C6	0.2636 (2)	0.13973 (11)	0.41112 (9)	0.0351 (3)	
C7	0.1992 (3)	0.36003 (11)	0.44683 (10)	0.0390 (4)	
H1	0.48184	0.44981	0.34877	0.0632*	
H1A	−0.032 (4)	0.3969 (16)	0.5380 (14)	0.0606*	
H1B	0.028 (3)	0.2603 (18)	0.5307 (13)	0.0606*	
H3	0.74118	0.21799	0.24920	0.0552*	
H4	0.64399	0.01822	0.27341	0.0513*	
H6	0.14354	0.11839	0.45083	0.0421*	

### Atomic displacement parameters (Å^2^)

**Table:**

	*U*^11^	*U*^22^	*U*^33^	*U*^12^	*U*^13^	*U*^23^
O1	0.0632 (7)	0.0386 (5)	0.0644 (7)	−0.0088 (5)	0.0331 (5)	0.0035 (5)
O2	0.0678 (7)	0.0286 (5)	0.0737 (7)	0.0004 (4)	0.0362 (5)	0.0027 (4)
O3	0.0834 (8)	0.0346 (5)	0.0760 (8)	−0.0045 (5)	0.0481 (6)	0.0018 (5)
O4	0.0723 (7)	0.0372 (6)	0.0751 (8)	0.0129 (5)	0.0267 (6)	−0.0080 (5)
N1	0.0732 (8)	0.0274 (6)	0.0610 (8)	0.0076 (5)	0.0389 (7)	0.0020 (5)
N2	0.0535 (7)	0.0333 (6)	0.0433 (6)	0.0050 (5)	0.0156 (5)	−0.0021 (4)
C1	0.0383 (6)	0.0321 (6)	0.0378 (6)	0.0010 (5)	0.0135 (5)	0.0011 (5)
C2	0.0412 (6)	0.0381 (7)	0.0411 (7)	−0.0044 (5)	0.0143 (5)	0.0029 (5)
C3	0.0454 (7)	0.0500 (8)	0.0490 (8)	−0.0033 (6)	0.0254 (6)	−0.0015 (6)
C4	0.0435 (7)	0.0433 (7)	0.0458 (7)	0.0044 (5)	0.0199 (5)	−0.0056 (5)
C5	0.0407 (6)	0.0315 (6)	0.0389 (7)	0.0024 (5)	0.0133 (5)	−0.0015 (5)
C6	0.0390 (6)	0.0319 (6)	0.0382 (6)	0.0023 (5)	0.0170 (5)	0.0007 (5)
C7	0.0451 (7)	0.0298 (6)	0.0452 (7)	0.0008 (5)	0.0161 (5)	0.0002 (5)

### Geometric parameters (Å, °)

**Table:**

O1—C2	1.3427 (17)	C1—C2	1.4167 (19)
O2—C7	1.2535 (16)	C1—C6	1.3935 (17)
O3—N2	1.2321 (15)	C2—C3	1.403 (2)
O4—N2	1.2210 (15)	C3—C4	1.376 (2)
O1—H1	0.8200	C4—C5	1.3914 (19)
N1—C7	1.324 (2)	C5—C6	1.3811 (17)
N2—C5	1.4615 (16)	C3—H3	0.9300
N1—H1A	0.914 (19)	C4—H4	0.9300
N1—H1B	0.88 (2)	C6—H6	0.9300
C1—C7	1.4896 (18)		
			
C2—O1—H1	109.00	C3—C4—C5	118.70 (13)
O3—N2—C5	117.94 (10)	N2—C5—C4	119.47 (11)
O4—N2—C5	119.21 (10)	N2—C5—C6	118.23 (10)
O3—N2—O4	122.86 (11)	C4—C5—C6	122.30 (12)
C7—N1—H1A	117.9 (12)	C1—C6—C5	119.72 (10)
C7—N1—H1B	121.1 (11)	O2—C7—C1	119.41 (13)
H1A—N1—H1B	120.8 (16)	N1—C7—C1	119.83 (11)
C6—C1—C7	122.57 (11)	O2—C7—N1	120.76 (13)
C2—C1—C6	118.51 (11)	C2—C3—H3	120.00
C2—C1—C7	118.91 (11)	C4—C3—H3	120.00
O1—C2—C3	117.79 (13)	C3—C4—H4	121.00
C1—C2—C3	120.33 (12)	C5—C4—H4	121.00
O1—C2—C1	121.89 (12)	C1—C6—H6	120.00
C2—C3—C4	120.43 (14)	C5—C6—H6	120.00
			
O3—N2—C5—C4	−171.90 (12)	C2—C1—C7—N1	−172.50 (13)
O3—N2—C5—C6	8.36 (17)	C6—C1—C7—O2	−170.64 (13)
O4—N2—C5—C4	8.18 (18)	C6—C1—C7—N1	9.1 (2)
O4—N2—C5—C6	−171.57 (12)	O1—C2—C3—C4	−179.42 (14)
C6—C1—C2—O1	178.56 (12)	C1—C2—C3—C4	0.7 (2)
C6—C1—C2—C3	−1.51 (19)	C2—C3—C4—C5	0.4 (2)
C7—C1—C2—O1	0.12 (19)	C3—C4—C5—N2	179.58 (12)
C7—C1—C2—C3	−179.95 (15)	C3—C4—C5—C6	−0.7 (2)
C2—C1—C6—C5	1.28 (17)	N2—C5—C6—C1	179.54 (11)
C7—C1—C6—C5	179.66 (12)	C4—C5—C6—C1	−0.20 (18)
C2—C1—C7—O2	7.73 (19)		

### Hydrogen-bond geometry (Å, °)

**Table:**

*D*—H···*A*	*D*—H	H···*A*	*D*···*A*	*D*—H···*A*
O1—H1···O2	0.82	1.79	2.5196 (16)	148
N1—H1A···O2^i^	0.914 (19)	1.969 (19)	2.8807 (17)	174.9 (18)
N1—H1B···O3^ii^	0.88 (2)	2.167 (19)	3.0193 (17)	164.6 (15)
C4—H4···O1^iii^	0.93	2.49	3.3915 (18)	164
C6—H6···O3^ii^	0.93	2.47	3.3826 (16)	169

Symmetry codes: (i) −*x*, −*y*+1, −*z*+1; (ii) −*x*, −*y*, −*z*+1; (iii) −*x*+3/2, *y*−1/2, −*z*+1/2.
